# Nuclear magnetic resonance investigation of water accessibility in cellulose of pretreated sugarcane bagasse

**DOI:** 10.1186/s13068-014-0127-5

**Published:** 2014-09-10

**Authors:** Jefferson Esquina Tsuchida, Camila Alves Rezende, Rodrigo de Oliveira-Silva, Marisa Aparecida Lima, Marcel Nogueira d’Eurydice, Igor Polikarpov, Tito José Bonagamba

**Affiliations:** Instituto de Física de São Carlos, Universidade de São Paulo, Caixa Postal 369, CEP 13560-970 São Carlos, SP Brazil; Current Address: Departamento de Engenharia de Materiais, Universidade Federal de São Carlos, Laboratório de Materiais Vítreos, Rod. Washington Luis, km 235, São Carlos, SP Brazil; Current Address: School of Petroleum Engineering, University of New South Wales, Building H6, Tyree Energy Technologies Building, Sydney, NSW 2052 Australia

**Keywords:** Sugarcane bagasse, Bioethanol, Acid pretreatment, Alkali pretreatment, Chemical composition, Solid-state NMR, Scanning electron microscopy

## Abstract

**Background:**

Enzymatic hydrolysis is a crucial step of biomass conversion into biofuels and different pretreatments have been proposed to improve the process efficiency. Amongst the various factors affecting hydrolysis yields of biomass samples, porosity and water accessibility stand out due to their intimate relation with enzymes accessibility to the cellulose and hemicellulose fractions of the biomass. In this work, sugarcane bagasse was subjected to acid and alkali pretreatments. The changes in the total surface area, hydrophilicity, porosity and water accessibility of cellulose were investigated by scanning electron microscopy (SEM) and nuclear magnetic resonance (NMR).

**Results:**

Changes in chemical and physical properties of the samples, caused by the partial removal of hemicellulose and lignin, led to the increase in porosity of the cell walls and unwinding of the cellulose bundles, as observed by SEM. ^1^H NMR relaxation data revealed the existence of water molecules occupying the cores of wide and narrow vessels as well as the cell wall internal structure. Upon drying, the water molecules associated with the structure of the cell wall did not undergo significant dynamical and partial moisture changes, while those located in the cores of wide and narrow vessels kept continuously evaporating until reaching approximately 20% of relative humidity. This indicates that water is first removed from the cores of lumens and, in the dry sample, the only remaining water molecules are those bound to the cell walls. The stronger interaction of water with pretreated bagasse is consistent with better enzymes accessibility to cellulose and higher efficiency of the enzymatic hydrolysis.

**Conclusions:**

We were able to identify that sugarcane bagasse modification under acid and basic pretreatments change the water accessibility to different sites of the sample, associated with both bagasse structure (lumens and cell walls) and hydrophilicity (lignin removal). Furthermore, we show that the substrates with increased water accessibility correspond to those with higher hydrolysis yields and that there is a correlation between experimentally NMR-measured transverse relaxation times and the efficiency of enzymatic hydrolysis. This might allow for semiquantitative estimates of the enzymatic hydrolysis efficiency of biomass samples using inexpensive and non-destructive low-field ^1^H NMR relaxometry methods.

## Background

Sugarcane bagasse is a residue from sugarcane milling for sugar and alcohol production and represents an abundant feedstock available to be converted into second generation biofuels [1,2]. In Brazil almost 600 million tons of sugarcane are processed every year [3], and 30% of this amount corresponds to bagasse. This material could be processed *in situ*, yielding cellulosic ethanol and other chemicals, by adapting the current milling plants already used for ethanol production within the framework of a biorefinery.

The classical approach to the production of cellulosic ethanol involves three key steps: 1) pretreatment of the feedstocks to decrease biomass recalcitrance, 2) enzymatic hydrolysis to break the carbohydrates into hexoses and/or pentoses and 3) sugar fermentation into alcohol [4,5].

Biomass recalcitrance is the major barrier to the industrial implementation of this process on a large scale since it decreases the efficiency of enzymatic hydrolysis and requires higher enzyme loading [4,6]. Thus, significant research efforts have been applied to decrease enzyme costs, to develop more efficient pretreatments and to understand the chemical and structural changes taking place as a consequence of different pretreatment technologies [1,7-9].

A variety of pretreatment methods have been proposed to decrease the recalcitrance of lignocellulosic matrices and to improve hydrolysis efficiency. The most common processes include milling [10,11], hot water and/or steam explosion [4,12], ammonia explosion (AFEX) [13], supercritical fluids [14,15], sulfite [16,17], diluted acids and bases [1,18-20] and irradiation [8,21].

Different pretreatments may have diverse effects on the biomass structure and chemical composition. The pretreatments may act, for instance, by promoting the decrease of cellulose crystallinity and/or degree of polymerization, by changing the lignin to hemicellulose ratio or by altering the total surface area of the substrate [1,2,8].

Lignin rearrangements, mainly characterized by its removal from the inner parts of the cell wall and redeposition on the surface, was described for other lignocellulosic biomasses submitted to steam explosion [22], diluted acid [23,24] and organosolv pretreatments [25].

Among the various factors that affect the rate of enzymatic digestibility, the modification of sample porosity was identified as one of the most important, because it directly influences the enzyme access to the substrate [6,26,27]. Due to the intimate contact between the cellulose and the enzymes that is required for the hydrolytic action to take place, the overall surface area, hydrated and accessible to the enzyme action, assumes a fundamental role to the process efficiency.

A variety of analytical techniques have been used to estimate the water accessibility in cellulose and the total surface area available in different lignocellulosic matrices. These techniques may use probing molecules, such as dextran in solute exclusion methods, or water in differential scanning calorimetry (DSC) and nuclear magnetic resonance (NMR) relaxometry [27-30]. They can also be based on the adsorption of a given molecule to lignocellulosic substrates, for instance, nitrogen adsorption to pore surfaces (BET method), proteins and enzymes adsorption, and the adsorption of dyes with a specific affinity to cellulose domains (Simon’s staining method) [6,26,31].

Tanaka *et al*. [26] compared the efficiency of enzymes with different sizes (cross-linked or normal cellulases) to deconstruct microcrystalline and amorphous cellulose. They observed that synergetic effects and hydrolysis yields are favored by the presence of pores on the substrate which are sufficiently large to allow the enzyme to diffuse in. Suurnäkki*et al*. [31] concentrated on the porosity profile resulting from enzyme action on pine and birch kraft pulps using solute exclusion and NMR techniques.

A relevant question in this field is how the different pretreatments affect the sample surface area available to hydrolysis, which could create several cellulose sites containing water molecules with varying mobility. Several published studies focus on the application of the techniques currently available for porosity determination to evaluate structural changes concerning porous distribution and cellulose accessibility on pretreated samples [6,27,30].

Wood samples undergoing thermomechanical, organosolv and steam treatments were studied by Chandra *et al*. [6], who estimated the cellulose accessibility on these samples using a modified version of Simons’ stain (SS) method. The method is based on a dye mixture containing direct blue (DB) and direct orange (DO) dyes and on their different sizes and cellulose affinities. This allowed the authors to establish a correlation between hydrolysis yields reached by each treatment and the ratio between the adsorbed amount of DO and DB [6]. Wood samples treated with dilute acid were also studied by NMR relaxation measurements, showing the potential of this technique to reveal the pore expansion within the plant cell wall [27].

In the present work, we used ^1^H NMR relaxometry and wide-line spectroscopy measurements to address this issue on sugarcane bagasse samples undergoing a two-step pretreatment. First, samples were treated with diluted sulfuric acid, and subsequently, with sodium hydroxide solutions of increasing concentrations. Dilute sulfuric acid pretreatment in the conditions applied in present work is known to depolymerize and solubilize hemicellulose fraction, whereas alkaline pretreatment results in substantial removal of lignin from the residual lignocellulose [1,5,8,10,17,18,20]. Therefore, the two-step pretreatment applied here aimed to separate the pretreatment step focused in hemicellulose removal from the one mostly impacting the lignin fraction of the biomass. In our previous publication [1], this pretreatment method has proved to be very efficient to improve hydrolysis yields on sugarcane bagasse samples. It removed up to 96% of hemicellulose and 85% of lignin and modified the cell wall microstructure by forming voids and separating cellulose bundles, thus improving water and enzyme access.

Herein, we used NMR and focused on the physicochemical changes of sugarcane bagasse due to pretreatments. In particular, we concentrate on the occurrence of cellulose sites presenting different pore sizes, associated with lumens and smaller pores inside the cell wall, and interactions with water molecules. For this purpose, we measured ^1^H NMR spectra and transverse relaxation times (*T*_2_), which depend on the physicochemical features of cellulose sites where water molecules can be accommodated [32-34]. Water molecules are found spatially distributed throughout the lignocellulosic matrix and can be thus used as probes to investigate cellulose accessibility and hydrophilicity. In order to better understand the interaction between water molecules and cellulose sites, ^1^H NMR measurements were performed under different degrees of samples hydration. Since the observed ^1^H NMR signal is a combined contribution from water and cellulose molecules, the most important NMR data for this work are those obtained from samples with higher hydration levels, where water signal predominates. The consistent decrease of the ^1^H NMR signal as a function of sample dehydration is the most important feature of the acquired data. This indicates that water molecules signal is being observed, as only these molecules are expected to be removed from the sample. For lower hydration levels (<10%) it is expected to have a higher overlapping of water and cellulose signals, making it more difficult to interpret ^1^H NMR data.

## Results

### Chemical composition

The chemical composition (cellulose, hemicellulose and lignin amounts) for all the bagasse samples is given in Table 1. Percentages of components are calculated on a dry weight basis, discounting amount of ash present in each sample. The latter biomass component was determined as the remaining inorganic fraction after the bagasse sample had been carbonized in a muffle.

**Table 1:** **Chemical composition of untreated bagasse and samples that underwent acid and alkali pretreatments**

**Bagasse samples**	**Bagasse composition (%)**			
	**Cellulose**	**Hemicellulose**	**Lignin**	**Total**
**Untreated bagasse**	45 ± 1	31.0 ± 0.9	28 ± 1	104 ± 4
**H**_**2**_ **SO**_**4**_ **1%**	58.3 ± 0.8	8.9 ± 0.1	33.6 ± 0.1	101 ± 2
**NaOH 0.25%**	68.3 ± 0.6	5.4 ± 0.1	26.1 ± 0.3	99.7 ± 0.9
**NaOH 0.5%**	72 ± 6	3.5 ± 0.1	24 ± 6	98.7 ± 0.4
**NaOH 1%**	83.2 ± 0.3	3.2 ± 0.1	11.2 ± 0.8	98 ± 1
**NaOH 2%**	85.8 ± 0.3	3.3 ± 0.1	9.6 ± 0.5	99 ± 1
**NaOH 3%**	87.3 ± 0.1	3.2 ± 0.1	9.7 ± 0.5	100.1 ± 0.4
**NaOH 4%**	85 ± 4	3.2 ± 0.1	9.4 ± 0.4	98 ± 5

All the samples treated with the different NaOH concentrations were previously treated with 1% H_2_SO_4_.

Values for cellulose include glucose, cellobiose and hydroxymethylfurfural amounts quantified by high performance liquid chromatography (HPLC). Hemicellulose comprises xylose, arabinose, furfural, glucuronic and acetic acids, while concentrations of soluble and insoluble lignins are added up to give the total lignin amount. Mass closure was obtained by adding cellulose, hemicellulose and lignin for each sample, and its total value is given in Table 1.

Before the pretreatments, bagasse without ashes comprised 45% cellulose, 31% hemicellulose and 28% lignin, as shown in the first row of Table 1. The cellulose amount increased continuously with the acid/base pretreatments up to between 85 and 87% under pretreatments using high NaOH concentrations (2% or higher) in the second step. It is important to notice that all the samples treated with sodium hydroxide were previously treated with sulfuric acid in the first pretreatment step. Most of the hemicellulose fraction was removed in the first acid step, as shown by its percentage decrease from about 31.0 to 8.9% (Table 1). The hemicellulose was further removed during subsequent alkaline steps, reaching minimum values around 3.2% for treatments with NaOH 0.5% or higher. Finally, the lignin relative percentage in bagasse increased slightly with acid pretreatment due to the removal of other components (mainly hemicelluloses) and then decreased progressively with pretreatments using NaOH concentrations between 0.5 and 2%.

Sodium hydroxide concentrations lower than 1% are very efficient at the removal of lignin and hemicellulose from bagasse samples. Alkali pretreatments with the NaOH concentrations higher than 2% become progressively less efficient and promote undesirable degradation and removal of cellulose fraction [1].

### Morphological changes during pretreatment

**Figure 1:**
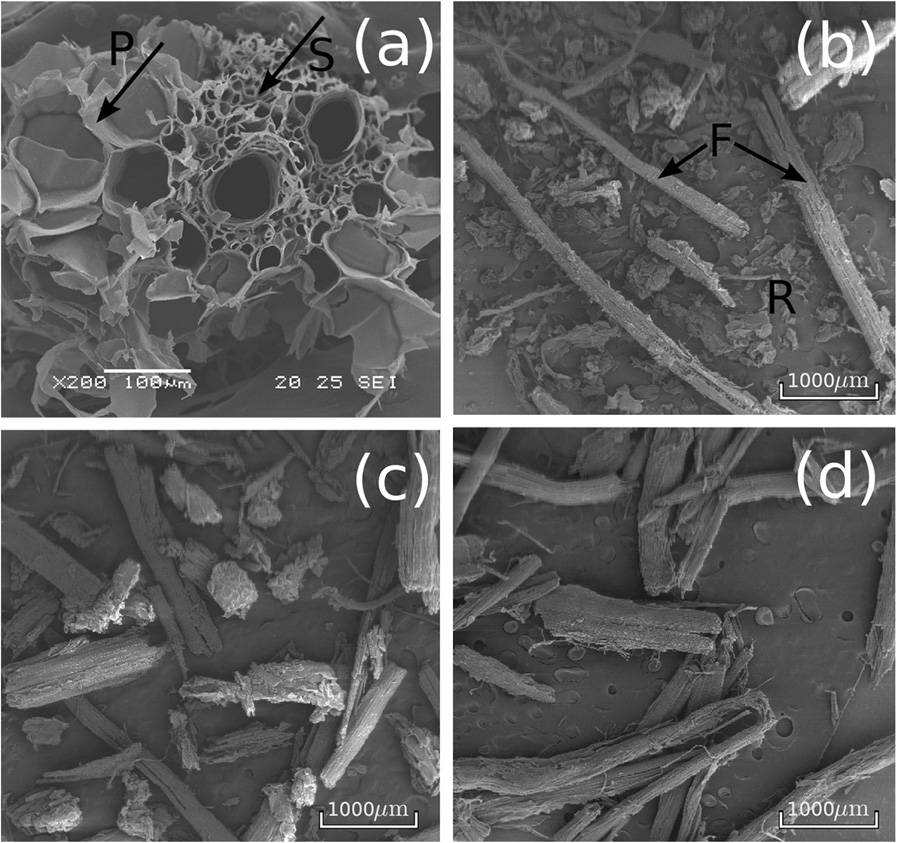
**Scanning electron microscopy images of the sugarcane bagasse. (a)** untreated bagasse, transversal section showing conducting vessels reinforced by sclerenchyma (S) and surrounded by parenchyma (P); **(b)** untreated bagasse, general view of the sample after milling, showing fibers (F) separated from the residues (R); **(c)** milled bagasse treated with acid, showing that the residues are significantly reduced after the first pretreatment step; **(d)** milled bagasse treated with acid and NaOH 0.5%, highlighting the predominance of fiber.

Sugarcane bagasse comes from sugarcane milling and the bagasse morphology is thus very similar to that of the internode region of the sugarcane stem. The tissue of the internode is basically formed by vascular bundles, surrounded by sclerenchymatous cells and embedded in parenchyma [35,36]. These features are shown in Figure 1a, where the index S indicates the sclerenchymatous tissue and P indicates the parenchyma cells. The number of vascular bundles increases from the center to the borders of the stem, so that at the outermost region of the stem they form a solid ring, while the center region generally becomes pithy.

Parenchyma cells, which form a soft filling tissue, are roughly separated from the conducting vessels and from the sclerenchyma during the milling process. After milling, conducting bundles reinforced by sclerenchyma result in more lignified and resistant sugarcane fibers, such as the ones indicated by F in Figure 1b, while the more fragile tissues constitute the pith residual component, as indicated by R in Figure 1b.

The relatively high amount of pith residues initially observed on the milled untreated bagasse is significantly decreased as a consequence of the pretreatments. Figure 1c shows a sample that underwent acid treatment (one step), whereby the predominance of bagasse fibers can be observed with only a small amount of pith. On samples that underwent the two-step pretreatment (acid followed by the alkaline step), only fibers can be observed in effect, as shown in Figure 1d for a sample treated with NaOH 0.5%. Residues are thus eliminated even when the lowest concentrations of sodium hydroxide are applied, which results in one important morphological difference between the untreated bagasse sample, the sample treated with acid only, and the samples treated with acid and base.

**Figure 2:**
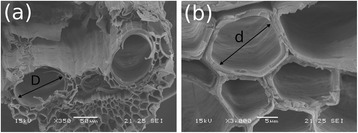
**Cross-section images of the sugarcane bagasse fibers obtained by scanning electron microscopy** **. (a)** fiber that underwent acid treatment, showing conducting vessels with two different diameter sizes; **(b)** amplification on a region of narrower diameters. The indexes D and d indicate wider and narrower diameters of approximately 70 and 10 μm, respectively.

The cross sections of these bagasse fibers show that their conducting vessels contain lumens with two distinct size distributions: two lumens with a wider diameter, indicated by D in Figure 2a, and many lumens with narrower diameters. Figure 2b shows a magnification of the region containing smaller lumens in a sample treated with acid where a smaller diameter (d) is indicated.

By analyzing a large number of different images and samples (treated and untreated bagasse), the wider lumen diameter (D) had an average value of approximately 70 μm (72 ± 10 μm), while the narrower lumen diameter (d) had an average value of about 10 μm (11 ± 5 μm). Cell wall boundaries around the lumens were 2 to 5 μm thick.

**Figure 3:**
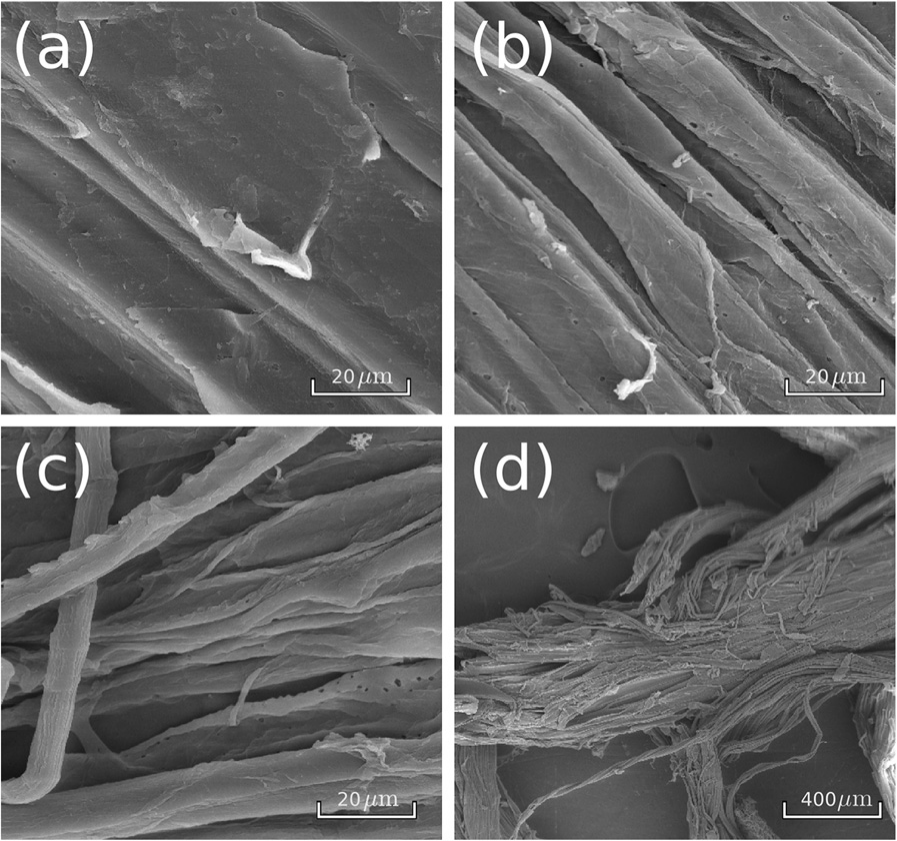
**Scanning electron microscopy images of the surface of sugarcane bagasse fibers before and after undergoing alkaline treatments. (a)** untreated sample, showing the closely packed structure of the fiber bundle surface; **(b)** sample treated with H_2_SO_4_ 1% and NaOH 0.5%, with individual fibers starting to come apart; **(c)** sample treated with H_2_SO_4_ 1% and NaOH 2%, with unattached and independent fibers and **(d)** general view of a degraded bagasse bundle with loose fibers.

Alkaline pretreatments have two important effects on the morphology of the bagasse fibers. First, the stiff structure of the fiber bundle is disturbed by the NaOH action, since the fibers start to detach from the neighboring fibers even at low sodium hydroxide concentrations (below 0.5%). As it can be observed in Figure 3a, prior to alkali pretreatment, the bundle surface has a compact structure with fibers closely packed together, while after being treated with acid and NaOH 0.5% the topology of individual fibers becomes more apparent (Figure 3b).

Under higher NaOH contents, the bundles become even more unstructured and may present completely independent fibers in some areas, as shown in Figure 3c for a sample treated with NaOH 2%. Figure 3d shows a general view of a sample treated under the same conditions, showing a bundle formed by loose fibers.

**Figure 4:**
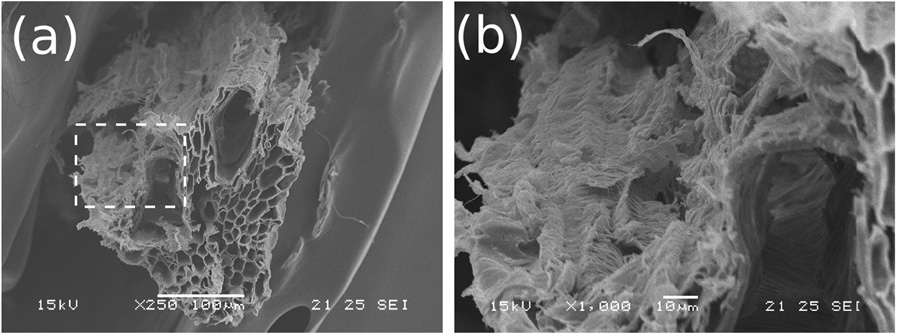
**Scanning electron microscopy images of the sugarcane bagasse treated with H**_**2**_ **SO**_**4**_ **1% and NaOH 1%. (a)** general view of the cross section of a fiber bundle after pretreatment and **(b)** amplification on the cell wall (dashed square in **(a)**), showing the surface damaged as a consequence of the pretreatments.

In addition to the changes in the bundle structures, alkali action has also modified the internal assembly of the cell walls. In Figure 4a, the cross-section of a sample treated with NaOH 1% is presented, showing the very fragile aspect of the bundle in comparison to a sample imaged before the alkaline treatment (Figure 2a, for instance). Amplification of the cell wall region reveals voids and a damaged structure. Both effects of the alkaline treatments must increase the sample total surface and the cellulose accessibility by liquid media and enzymes.

### Nuclear magnetic resonance relaxation

**Figure 5:**
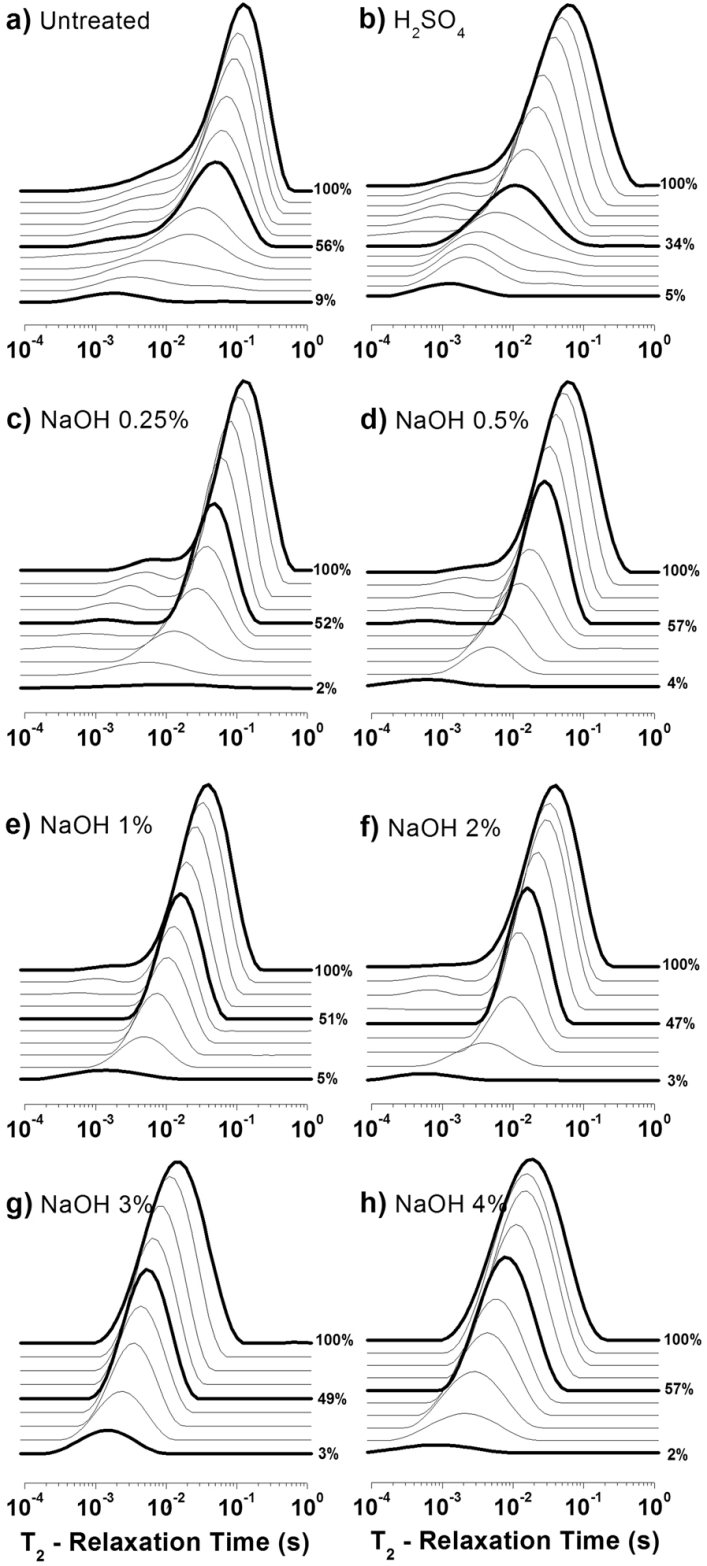
***T***_**2**_ **-distributionsversusrelative humidities for bagasse samples. (a)** Untreated, **(b)** H_2_SO_4_ 1%, **(c)** NaOH 0.25%, **(d)** NaOH 0.5%, **(e)** NaOH 1%, **(f)** NaOH 2%, **(g)** NaOH 3%,**(h)** NaOH 4%. Darker lines were included only to indicate extreme and intermediate hydration levels. All the samples treated with NaOH were previously treated with H_2_SO_4_ 1%.

NMR relaxation data were analyzed by Inverse Laplace transform (ILT), resulting in distributions of transverse relaxation times (*T*_2_-distributions) [37-39]. Figure 5 shows the *T*_2_-distributions obtained for bagasse samples that were submitted to the drying procedure being stack plotted for different relative humidities. Every set of curves corresponds to a different pretreatment.

The *T*_2_-distributions were fitted by log-Gaussian functions as described in the experimental section, allowing free adjustment of all the function parameters. The fitting process was done by using the data obtained in the previous adjustment as initial parameters, starting from the highest humidity (100%) for each sample studied by NMR.

**Figure 6:**
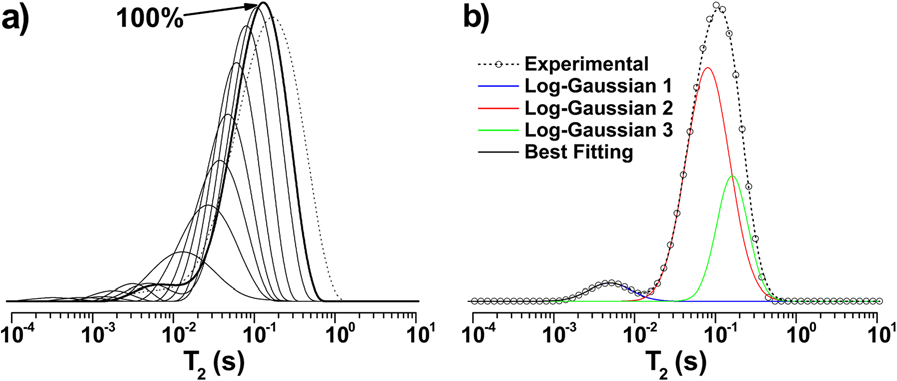
**Fitting procedure. (a)** Procedure to define the maximum relative humidity (100%) exemplified for the sample NaOH 0.25%. Dashed line represents *T*_2_-distribution for oversaturated sample; **(b)** Example of fitting using three log-Gaussian functions for sample NaOH 0.25% with relative humidity of 100%.

In order to define the highest humidity (100%), the excess of free water in the sample after oversaturation was taken into account. The excess water presented the longest observed *T*_2_ components and corresponded to an intense peak at the right side of *T*_2_-distributions. When the sample was oversaturated, the high intensity of this peak interfered with the observation of shorter components in the *T*_2_-distribution. The definition of the 100% humidity corresponds to the drying step in which the peak assigned to excess free water became the smallest contribution for the overall *T*_2_-distribution (Figure 6a, dashed line).

To the resulting *T*_2_-distributions, it was possible to assign three log-Gaussians for all the samples (see example in Figure 6b), except for the case of samples treated with NaOH 3% and NaOH 4%, for which we assigned only two log-Gaussians. Although these log-Gaussians may overlap each other along the *T*_2_-distributions; this fitting procedure allows us to quantitatively estimate how each region of the *T*_2_-distribution varies as a result of water saturation [37-39].

The log-Gaussian *T*_2_distributions were associated with three sites with different water affinities along the sample, corresponding to large, intermediate and small average transverse relaxation (*T*_2M_) values. These were assigned to wide and narrow lumens (diameters D and d in Figure 2, respectively), and to water within the porous structure of the cell wall (including the involucre of the lumens in Figures 2 and 4), respectively.

Water molecules located in the different sugarcane bagasse structures show *T*_2_-distributions ranging from longer to shorter components of *T*_2_. Longer *T*_2_ components were associated with higher mobility water molecules located in the core of the vessels with two different diameter sizes (Figure 2), while shorter *T*_2_ components were associated with water molecules with lower mobility, sorbed at the cell wall around the lumens. The water molecules with shorter *T*_2_ components had their mobility reduced due to the intense hydrophilic interactions. In fact, water molecules in the sugarcane bagasse matrix showed a continuous dynamics distribution, ranging from low mobility molecules (cellulose-sorbed water molecules) to very mobile ones, however not reaching bulk water dynamics. This was a consequence of the small diameters of the conducting plant lumens (about 10 and 70 μm), which caused the water molecules to experience the mobility restriction imposed by the cellulose surfaces. In the case of the sample treated with NaOH 0.25%, for example, three log-Gaussians could be identified when this sample was fully hydrated (100%; Figure 6).

Continuous drying of the sample introduced a steady decrease in the *T*_2M_ values associated with the water molecules within the wide and narrow vessels, until it reached considerably small values (at around 20% humidity), comparable to those observed for water confined within the cell wall (at humidity equal to 100%). These results suggest that the more mobile water molecules are easily removed during the initial steps of the drying procedure and, at the end of the process, only water molecules contained within the cell wall around the lumens remain in the vessels. This is the region associated with the cellulose surface.

**Figure 7:**
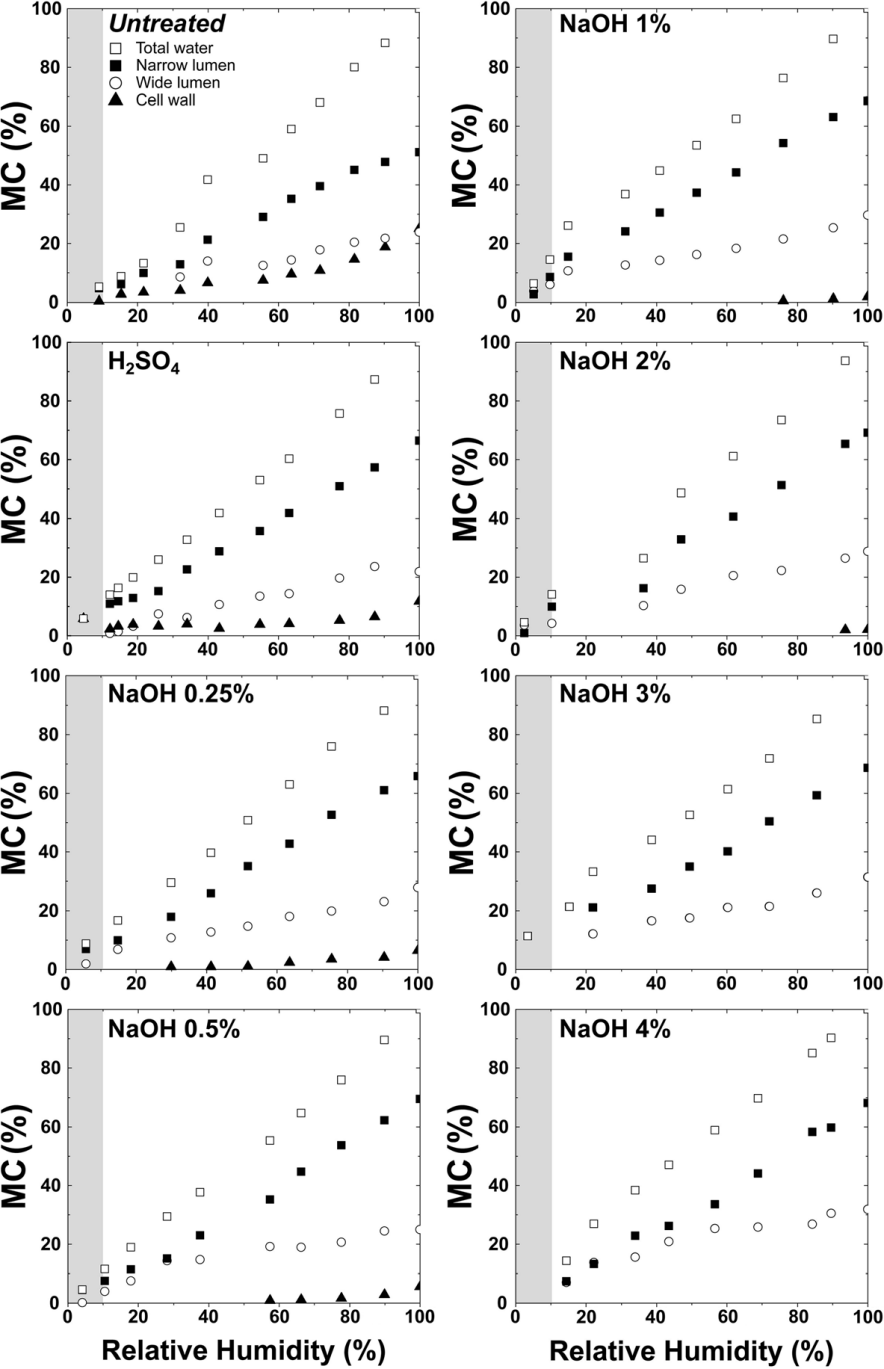
**Evolution of total and partial moisture content (MC) versusrelative humidity for bagasse samples treated under different conditions.**

**Figure 8:**
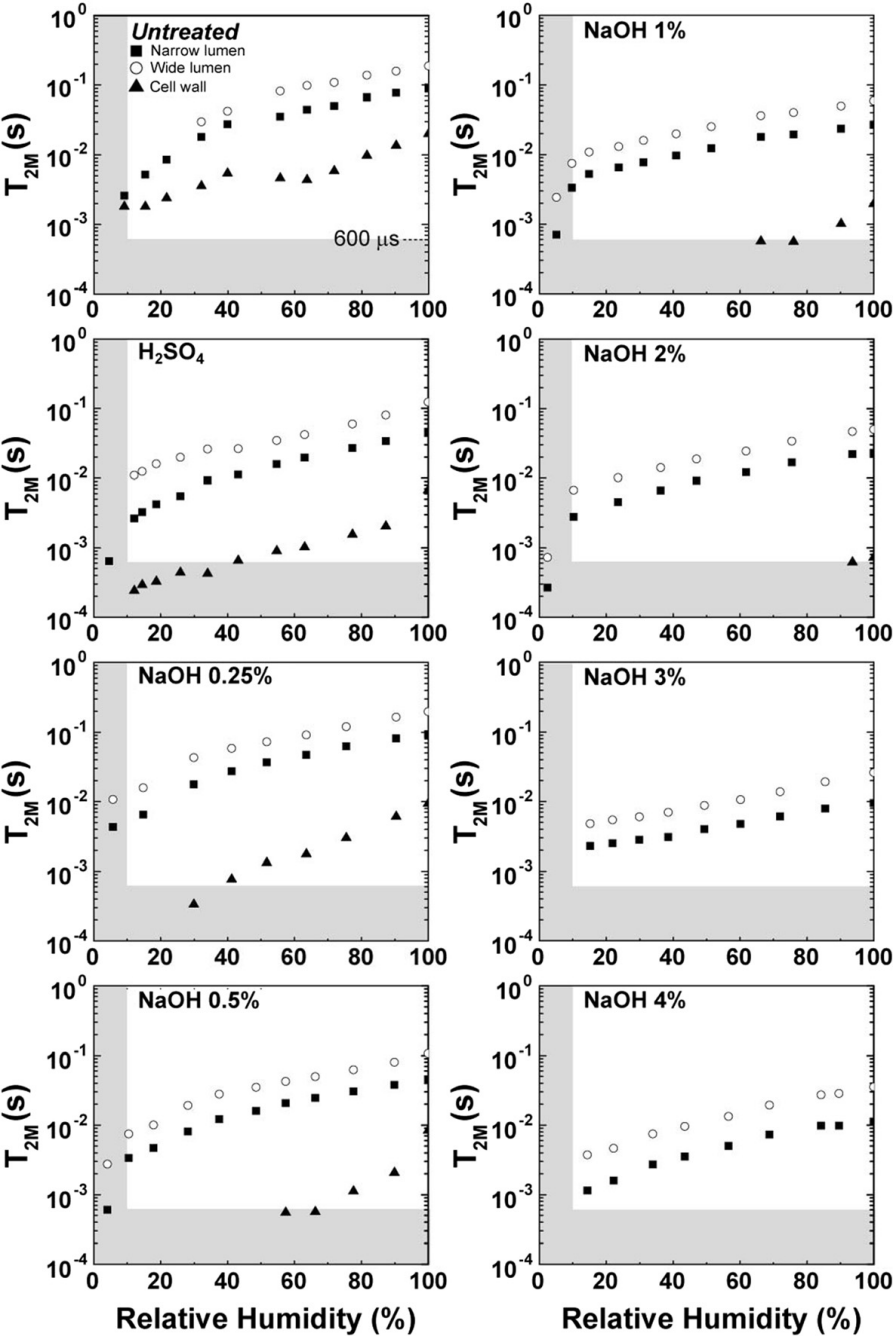
***T***_**2M**_ **versus relative humidity for bagasse samples treated under different conditions.**

Figures 7 and 8 show the total and partial moisture contents (MC) of bagasse samples and the average *T*_2M_ values versus relative humidity, respectively. Partial moisture contents refer to water from different environments (narrow and wide lumens and water associated with cellulose surface within the cell wall).

The vertical shaded areas in both Figures 7 and 8 indicate the measurements for which *T*_2M_ and MC are very difficult to estimate due to the very low amount of water (humidity lower than 10%) and very short relaxation times. The horizontal shaded area in Figure 8, including average relaxation times (*T*_2M_) of the order 600 μs or shorter, indicates a region where the ILT presents greater errors when obtaining the *T*_2_-distributions due to the signal sampling rate employed in the Carr-Purcell-Meiboom-Gill (CPMG) experiments [Carr1954,Meiboom1958]. Therefore, it is important to point out that only a few experimental points are presented in these regions because they are challenging to detect using NMR relaxation methods. In this region one can also expect to have superposition of water and cellulose ^1^H NMR signals. These data points are shown in Figure 8 just to indicate the ^1^H NMR water molecules *T*_2_ trends for low sample humidity. Additional information about water under these two conditions can be accessed by ^1^H NMR spectroscopy, which will be presented and discussed shortly.

From Figure 7, besides following the correlation between total and partial moisture contents (MC) and relative humidity, one can observe that water in the lumen cores is easier to remove than the water in the internal part of the cell wall. Comparison of the curves also reveals that the narrow lumens contain the highest amount of water.

The *T*_2M_ values measured for all regions of the sample tend to be shorter values when the relative sample humidity or moisture content decreased during the drying procedure (Figure 8). This result is expected since the water molecules within lumen cores are easier to remove by drying, while water molecules sorbed on the cell wall tend to remain in the sample. It is worth mentioning that the *T*_2M_ values observed for water molecules sorbed inside the walls (at 100% relative humidity) are similar to those measured for water molecules associated with narrow and wide lumens at relative humidities below 20%. This result indicates that the remaining water molecules, even in the lumens, are sorbed on the cell wall.

**Figure 9:**
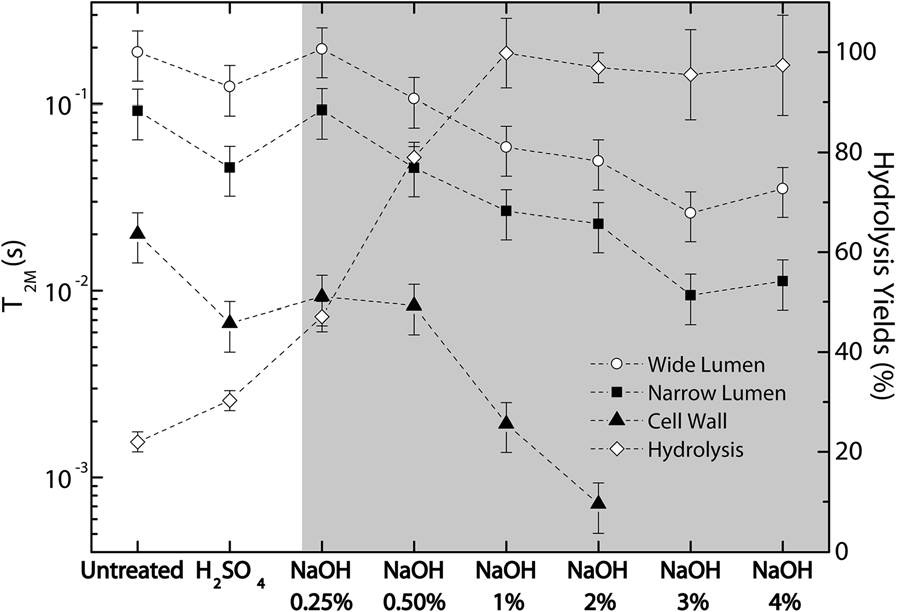
***T***_**2M**_ **values and hydrolysis yields versus pretreatment conditions for samples with 100% humidity.** All samples treated with NaOH were previously treated with H_2_SO_4_ 1%. Shaded area evidences the samples with NaOH treatment.

Taking into consideration samples with 100% humidity, one can observe (Figure 9) that the *T*_2M_ values tend to decrease as a function of the pretreatment, being smallest for the most concentrated NaOH solution (4%). This indicates that water molecules have, on average, lower dynamics for the samples treated under higher NaOH concentrations, which could present higher hydrophilicity due to continuous removal of lignin (see Table 1), the hydrophobic component of plant structure. Similar trends are observed for the other sample humidities. Figure 9 shows also the hydrolysis yields as a function of pretreatment steps, which reveals a correlation of the hydrolysis yields with *T*_2M_ values. This indicates that substrates with increased water accessibility correspond to those with higher hydrolysis yields, according to results of enzymatic digestibility previously reported for these samples.

### Nuclear magnetic resonance spectroscopy

**Figure 10:**
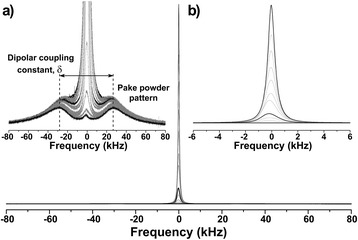
^**1**^ **H NMR spectra as a function of the drying procedure.** Botton spectra obtained for the bagasse sample treated with NaOH 3%. Zooms of **(a)** vertical and **(b)** horizontal axes. Darker lines were included only to indicate extreme (<1 and 100%) and intermediate (20%) hydration levels.

^1^H NMR spectra were obtained for all bagasse samples treated with NaOH. Figure 10 shows a typical spectra obtained following the drying procedure for the sample treated with NaOH 3%. Similar results were observed for all the other samples (not shown).

From Figure 10a one can directly identify at least two components in the spectra: a central lorentzian-like line relatively narrow (~kHz) and a broadened Pake powder pattern [40] with a dipolar coupling constant (δ) of about 50 kHz.

In the fitting analysis, it was possible to identify a third wide Gaussian spectral component of approximately 50 kHz, which was superimposed to the Pake powder pattern. The lorentzian-like line was assigned to more mobile water molecules located in the core of both large and small vessels, and also within the cell wall around the lumens, also observed in the relaxation measurements. As opposed to the case of relaxation measurements, mobile water molecules located in the core of both wide and narrow lumens, as well as inside the cell wall, could not be distinguished from the spectra (Figure 10). For this reason, *T*_2_ relaxation measurements are more appropriate for studying mobile water molecule dynamics.

Since the intensity of the Pake pattern remained practically constant during drying process, it could be assigned to both polycrystalline cellulose and/or water molecules strongly bound to the surface of polycrystalline cellulose microfibrils. Similar assignment could be attributed to the wide Gaussian component in the spectra, however, associating it with amorphous cellulose. The above assignments are corroborated by the fact that the cellulose samples presented crystallinities of about 70% [41], a percentage that can be obtained directly from the ratio of the signal observed for Pake and Gaussian spectral components for MC >10%. ^2^H NMR should be used for the correct study of water molecules dynamics under low humidity levels.

**Figure 11:**
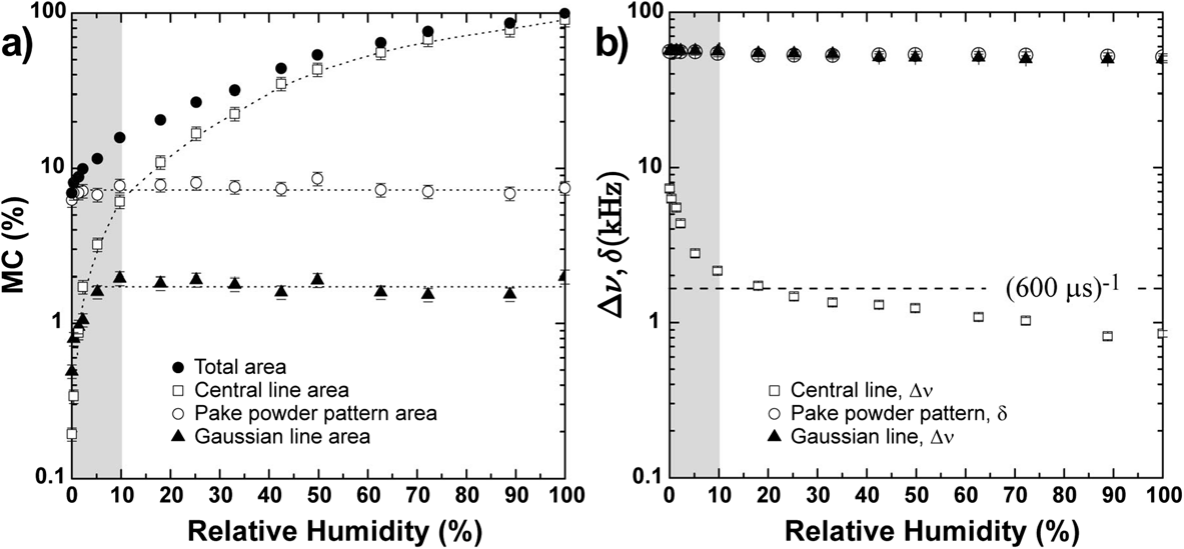
**Nuclear magnetic resonance spectroscopy results.** **(a)** Total and partial moisture content estimated from the^1^H NMR spectra; **(b)** line widths (Δν) and dipolar coupling (δ) as a function of relative humidity for the bagasse sample treated with NaOH 3%. Shaded areas in both plots indicate the regions where the quality of NMR relaxation measurements would be poor due to low signal-to-noise ratio.

It is important to mention that the strongly bound water molecules observed by NMR spectroscopy are not the same as observed by NMR relaxation, because they would present very short *T*_2_ components of the order of 1/δ, approximately 20 μs, far below the estimated shortest observable average relaxation time obtained by relaxation experiments (approximately 600 μs), as shown in Figure 11b. Having these three spectral components in mind, the line widths Δν (full with at half maxima) and dipolar coupling constant δ were estimated according to Figure 11.

As can be observed in Figure 11, mobile water molecules located in the core of both large and small vessels, as well as the waters contained within the cell wall, were relatively easy to remove by drying. Conversely, the water molecules putatively strongly bound to cellulose surfaces, sorbed on polycrystalline or amorphous cellulose structures, were practically irremovable. While the estimated partial moisture content of polycrystalline cellulose remained constant during the drying procedures, this parameter for amorphous cellulose started decreasing when relative humidity dropped below 10%. As already mentioned, this issue should be studied in detail by the use of ^2^H NMR. Efforts in this direction are being undertaken by our group.

## Discussion

The chemical pretreatments applied to sugarcane bagasse introduced changes in chemical and physical characteristics of this material. As can be observed in Table 1, the acid pretreatment removed most of the hemicellulose present in the samples. On the other hand, the second alkaline step of the pretreatment was mostly responsible for the removal of lignin (Table 1) and for the morphological changes in the cell wall around the lumens (Figures 3 and 4). The removal of these components changed the porosity of the cell wall on a scale observable by SEM, forming voids and opening the closed structure of the untreated bagasse fibers, as can be observed in Figure 4. NMR data confirmed the changes observed by SEM according the discussions below.

The log-Gaussian *T*_2_-distributions obtained indicated the existence of water molecules under three different degrees of mobility on sugarcane samples. The most mobile molecules, presenting longer *T*_2_ components, occupied the core of wide and narrow lumens. The most hindered ones, with the shortest *T*_2_ components, were located within the internal structure of the cell wall. The intermediate *T*_2_ values were associated with water molecules present in regions where a cooperative combination of high and low mobility defined the average values of the observed relaxation times.

**Figure 12:**
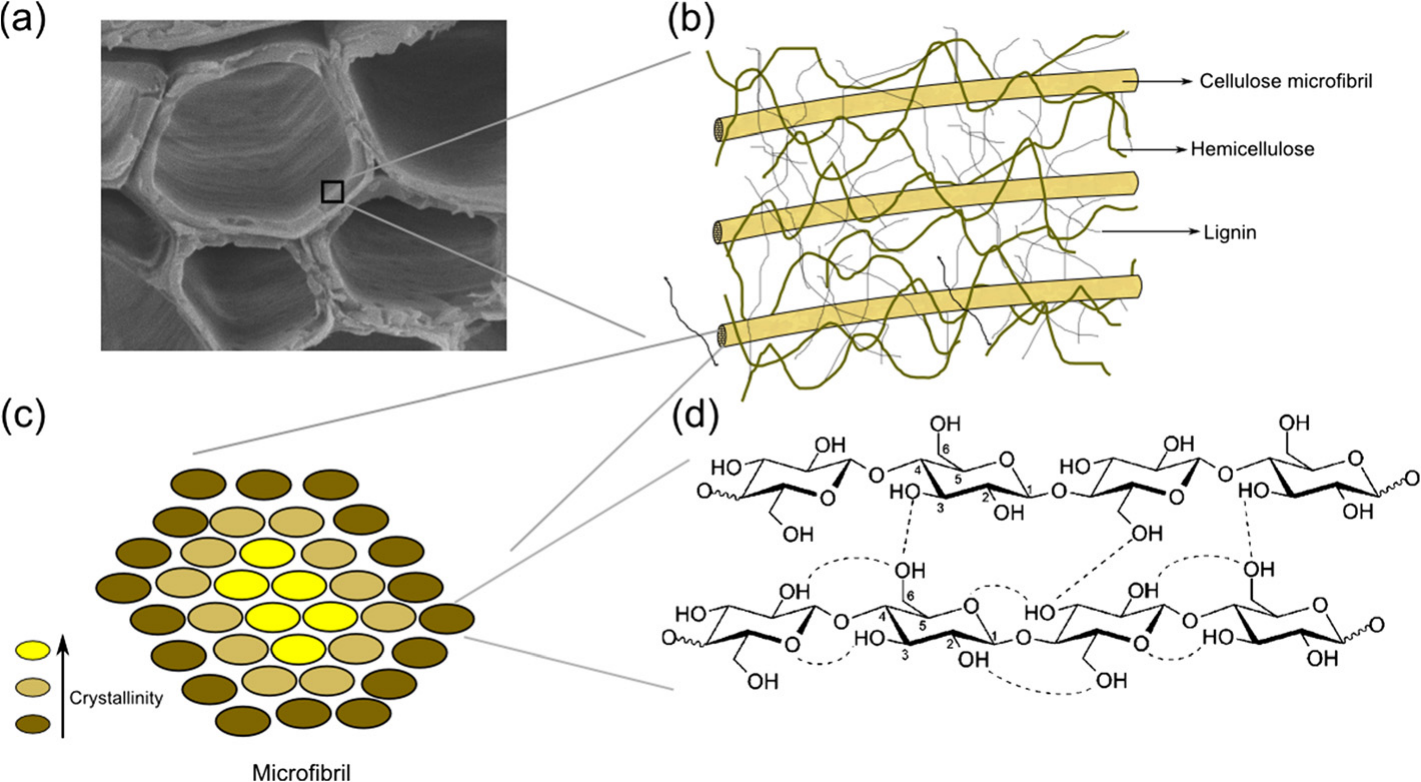
**Structure of the plant cell wall. (a)** Cross section of the bagasse cell wall, showing the involucre of the lumens; **(b)** schematic representation of the spatial distribution of the cell wall components and of the internal structure of the region amplified from the wall in **(a)**; **(c)** scheme of the transversal section of a cellulose microfibril; **(d)** cellulose fibrils are formed by cellulose polymeric chains.

The general model of the plant cell wall describes its composition as an entangled assemble formed by cellulose microfibrils immersed in a matrix of hemicellulose and cross-linked lignin [42]. The schematic representation of a model of spatial distribution of the cell wall components is shown in Figure 12. Part of the (more mobile) water molecules is contained inside the vacant lumens, shown in Figure 12a. On the other hand, the water molecules with more restricted mobility are contained within the cell wall surrounding the lumen (Figure 12a). A detailed picture of the cell wall (Figure 12b) shows that these waters would be permeating cellulose fibrils and the other components of the wall, establishing hydrophilic interactions. In all cases, water mobility differs from that observed on bulk molecules because even the more mobile water molecules contained in the lumens (diameters of about 10 and 70 μm) are more confined than they would be in the bulk, due to interactions with the cell wall surfaces.

The behavior of the water populations under the drying process is shown in Figures 7 and 8. While the *T*_2M_ values associated with cellulose fibrils did not change significantly during the drying procedure, the *T*_2M_ values from water in the wide and narrow vessels kept continuously decreasing until relative humidity was about 20%. This indicates that in the initial steps of the drying procedure water is first being removed from the cores of lumens and, at the end of the process, the only water molecules remaining in the samples are those located inside the cell walls under different degrees of mobility, with the most confined water populations non-covalently bound to cellulose fibers.

Besides the effect of pretreatments in increasing the porosity of the complex microstructure of the cell wall, the ratio of its hydrophilic to hydrophobic components is also being modified. In particular, lignin removal performed by alkali pretreatments promotes sample hydrophilicity, since lignin network is highly hydrophobic. This effect is probably responsible for the changes in *T*_2M_ values with the different pretreatments observed in Figure 9. The curves for the three distinct *T*_2M_ values associated with the different water environments show a steady decrease in *T*_2M_ times in relation to increases in NaOH concentrations, indicating increased hydrophilicity of the surfaces as a consequence of lignin removal. An interesting feature to be noticed is that the *T*_2M_ values associated with the cellulose surfaces inside the cell wall (triangles in Figure 9) decrease more rapidly with the NaOH pretreatments than the curves of *T*_2M_ values related to lumens. This indicates that these regions are severely modified by the pretreatments and that the conditions applied to decrease the cell wall hindrance of these samples are being efficient. The stronger interaction between the water molecules and the surface of the bagasse treated under alkaline conditions is in agreement with our previous results reported for the enzymatic digestibility of these samples [1]. Table 2 shows the yields of enzymatic hydrolysis after 48 hours of enzyme action obtained in bagasse samples treated under different conditions. The hydrolysis yield in each case is obtained by dividing the total amount of glucose liberated from a sample by its total cellulose content. As can be observed on Table 2 and in Figure 9, the hydrolysis efficiency is highly improved as the samples undergo the two-step pretreatment. While only 22% of the cellulose amount was converted to glucose in untreated bagasse, hydrolysis yields above 95% were obtained in samples treated under NaOH concentrations of 1% or higher. These results confirm the correlation between the pretreatment efficiency, the water interaction with the cell wall and the enzyme accessibility to these substrates. Since *T*_2M_ times are directly related with the general phenomenon involving improved interactions between water molecules and the cell wall components and their increased wettability, it is tempting to speculate that the same method can be applied for semiquantitative evaluations of the efficiency of enzymatic hydrolysis of the biomass samples subjected to other types of pretreatments. This might allow for the use of the inexpensive and non-destructive low-field 1H NMR relaxometry technique for semiquantitative predictions of the enzymatic hydrolysis efficiency of biomass samples.

**Table 2:** **Results for total hydrolysis yields obtained from bagasse samples after 48 hours under enzyme action** [1]

**Bagasse samples**	**Enzymatic hydrolysis (48 hours)**
	**Total hydrolysis yield, %**
**Untreated bagasse**	22.0 ± 0.3
**H**_**2**_ **SO**_**4**_ **1%**	30.3 ± 0.3
**NaOH 0.25%**	47.1 ± 0.9
**NaOH 0.5%**	79 ± 2
**NaOH 1%**	100 ± 7
**NaOH 2%**	97 ± 2
**NaOH 3%**	96 ± 10
**NaOH 4%**	97 ± 20

Hydrolysis yield values are expressed as an average (± standard deviation) of duplicate determination. All the samples treated with NaOH were previously treated with 1% H_2_SO_4_.

The two first points in each curve of Figure 9 shows the distinctive behavior of the *T*_2M_ values from untreated bagasse and from bagasse treated with H_2_SO_4_ when compared to the other samples that underwent alkaline pretreatments. This is probably associated with the different morphological characteristics of these samples. SEM images presented in Figure 1 show that these two samples are formed by two main features: bagasse fibers and pith residues. However, the samples that underwent the alkaline pretreatment contain practically only bagasse fibers. This fact indicates that the distinct behavior of these two samples might be related to the presence of residual material besides the lumen structures.

## Conclusions

Morphological changes of sugarcane bagasse samples, promoted by two-step pretreatments, change the water interaction and accessibility to different sites of the sugarcane cell wall. Based on the information obtained from SEM and NMR, we were able to identify water molecules located in the lumens core (high mobility) and in the internal part of the cell walls (low mobility).

Pretreatments also change the ratio of hydrophilic to hydrophobic components of the cell wall matrix, thus modifying interactions between water molecules and the different binding sites of the sample. The samples in which the water interaction with the bagasse cell wall is facilitated by the pretreatment are also the ones that present improved enzymatic hydrolysis yields. Our results show that non-destructive ^1^H NMR relaxometry might be used as an inexpensive and practical method for semiquantitative determination of the efficiency of enzymatic hydrolysis of plant biomass samples.

## Methods

### Materials

Grounded sugarcane bagasse was kindly provided by the Cosan Group (Ibaté, São Paulo, Brazil), and used as received, without further washing or milling. During the industrial milling process, bagasse was washed with hot water at 70°C to remove soluble sugars. Prior to pretreatments, grounded bagasse was passed through a 9.8 mm sieve to limit the maximum particle size and then dried in a convection oven at 60°C for 24 hours. Sulfuric acid and sodium hydroxide for sample pretreatments were purchased from JT Baker (Mexico City, Mexico) and from Mallinckrodt Chemicals (Linköping, Sweden), respectively, and were used as received.

### Bagasse pretreatments

Sugarcane bagasse was initially hydrolyzed with diluted H_2_SO_4_ (1% v/v in water) for 40 minutes at 120°C. The pressure was kept at 1.05 bar and a 1:10 solid to liquid ratio (gram of bagasse/ml of solution) was used. Bagasse solid fraction was separated from the hydrolysate by filtration and abundantly washed with tap water to eliminate acid excess before oven drying at 60°C for 24 hours. A second pretreatment step for bagasse delignification followed, using one of the following NaOH solutions with increasing concentrations (0.25, 0.5, 1.0, 2.0, 3.0 or 4.0% w/v), at 120°C for 40 minutes. Six pretreated bagasse samples were then obtained by filtration; washing until neutral pH is reached and drying of the solid in oven for 24 hours at 60°C. Enzymatic hydrolysis assays were carried out on pretreated bagasse samples, as described in [1].

### Chemical composition

The percentage amounts of cellulose, hemicellulose and lignin in bagasse were determined as described in detail elsewhere [1]. Raw bagasse was previously extracted in 95% ethanol and all the samples were milled until able to pass through a 2 mm sieve before undergoing total acid hydrolysis, using a 72% (v/v) H_2_SO_4_ solution. Solid fraction was separated from the hydrolysate by filtration though quantitative filter paper, rinsed until neutral pH and then oven dried at 105°C to a constant weight (containing insoluble lignin and ash). Ash content was then determined by calcination in a muffle (EDG 10PS, São Carlos-SP, Brazil) at 800°C for 2 hours, and used to determine the insoluble lignin amount by subtraction.

Soluble lignin was determined by absorbance measurements (280 nm) using a UV-VIS spectrophotometer (model Lambda 25, Perkin Elmer, Waltham, MA, USA), and taking into account the interfering absorption of furfural and hydroxymethylfurfural, as previously described [43].

The hydrolysate was also analyzed by HPLC to determine sugars, organic acids, furfural and hydroxymethylfurfural. HPLC determinations were performed in a Shimadzu LC-10 AD chromatograph (Shimadzu, Kyoto, Japan) equipped with refractive index and UV-VIS detectors (Shimadzu SPD-10, Kyoto, Japan). A detailed description of the columns, mobile phases and analytical conditions employed can be found in Rezende *et al*. [1].

### Nuclear magnetic resonance

^1^H NMR relaxation measurements were carried out using a LapNMR console (Tecmag, Houston, USA) and a permanent Bruker magnet (Bruker, Billerica, USA) operating at 0.47 T (20 MHz). Transverse relaxation times (T_2_) were measured using CPMG sequence [44,45], with π/2 radiofrequency (rf) pulses of 6.6 μs, delay between π pulses of 60 μs, and recycle delay of 15 seconds. NMR relaxation data were analyzed by ILT, resulting in a distribution of transverse relaxation times (*T*_2_-distributions). The *T*_2_-distributions were analyzed by least-squares fitting using logarithmic-Gaussian (log-Gaussian) functions, according to the expression:

1 $$ F\left(\tau \right)=A{e}^{-\frac{1}{2}{\left( \log \left(\frac{\tau }{T_{2M}}\right)/\sigma \right)}^2} $$

where *A* is proportional to the number of water molecules, which was used for estimating the total and partial moisture contents (MC) of bagasse samples, *T*_2M_ is the average transverse relaxation value, and σ is the full width at half maximum of the distribution.

^1^H NMR spectra were acquired using a Varian UNITY Inova spectrometer (Varian, Palo Alto, USA) operating at 8.22 T (350 MHz) and a 7-mm Jakobsen static probe (Varian, Palo Alto, USA). A single π/2 pulse sequence was used to obtain the free induction decays (FIDs) and respective spectra, with rf pulses of about 3.5 μs long and recycle delays of 7 seconds.

NMR experiments were carried out along a drying procedure consisting of submitting the wet samples to subsequent steps of 5-minutes drying under vacuum at 60°C. Samples were weighed before every NMR experiment, in order to evaluate hydration levels.

### Scanning electron microscopy

Bagasse morphology was analyzed by SEM before and after undergoing pretreatments. Samples from surfaces or transversal sections (obtained by fracture in liquid N_2_) were oven dried and coated with Au in a SCD 050 sputter coater (Oerlikon-Balzers, Balzers, Liechtenstein) Sample imaging was carried out using the scanning electron microscopes, models DSM 960 (Zeiss, Oberkochen, Germany) or JSM 5900LV (Jeol, Tokyo, Japan).

Sample features such as lumen diameters and cell wall thickness were manually measured using the program Axio Vision 4.8 (Carl Zeiss, Oberkochen, Germany). Averaged values of the lumen diameters were obtained by measuring about 350 lumens from different regions of raw bagasse and treated samples (four images by sample). Since most of the lumens have a distorted circumferential aspect, two diameters were measured: the maximum and the minimum axis, approximately perpendicular to each other, so that a mean diameter could be obtained for each lumen.

## Abbreviations

CPMG: Carr-Purcell-Meiboom-Gill
DSC: Differential scanning calorimetry
FID: Free induction decay
HPLC: High performance liquid chromatography
ILT: Inverse Laplace transform
MC: Moisture contents
NMR: Nuclear magnetic resonance
SEM: Scanning electron microscopy
*T*_2_: Transverse relaxation
*T*_2M_: Average transverse relaxation
